# The Effects of Sub-inhibitory Antibiotic Concentrations on *Pseudomonas aeruginosa*: Reduced Susceptibility Due to Mutations

**DOI:** 10.3389/fmicb.2021.789550

**Published:** 2021-12-20

**Authors:** Kay A. Ramsay, Sharla M. McTavish, Samuel J. T. Wardell, Iain L. Lamont

**Affiliations:** Department of Biochemistry, University of Otago, Dunedin, New Zealand

**Keywords:** *Pseudomonas aeruginosa*, antibiotic resistance, sub-inhibitory concentration, genetic mutations, resistance mechanisms, cystic fibrosis, sub-lethal concentration

## Abstract

*Pseudomonas aeruginosa* chronically infects in the lungs of people with cystic fibrosis and other forms of lung disease. Infections are treated with antibiotics, but over time, the bacteria acquire mutations that reduce their antibiotic susceptibility. The effects of inhibitory amounts of antibiotics in selecting for antibiotic-resistant mutants have been well studied. However, the concentrations of antibiotics that reach infecting bacteria can be sub-inhibitory and but may nonetheless promote emergence of antibiotic-resistant bacteria. Therefore, the aim of this research was to investigate the effects of sub-inhibitory concentrations of antibiotics on the antibiotic susceptibility of *P. aeruginosa*. Two *P. aeruginosa* reference strains, PAO1 and PA14, and six isolates from individuals with cystic fibrosis were studied. The bacteria were passaged in the presence of antibiotics (ceftazidime, ciprofloxacin, meropenem or tobramycin) at sub-inhibitory amounts. Fifteen populations of bacteria (up to five per strain) were exposed to each of the four antibiotics. Antibiotic susceptibility was determined following 10 passages on agar supplemented with antibiotic and compared with susceptibility prior to antibiotic exposure. Antibiotic exposure resulted in susceptibility being significantly (>2-fold) reduced for 13 of the 60 populations. Seven samples had reduced susceptibility to ciprofloxacin, three to tobramycin, two to ceftazidime and one to meropenem. Whole-genome sequencing revealed the mutations arising following antibiotic exposure. Mutants with reduced antibiotic susceptibility had mutations in genes known to affect antibiotic resistance, including regulators of efflux pumps (*mexR*, *mexS*, *mexZ* and *nalC*) and the *fusA1* gene that is associated with aminoglycoside resistance. Genes not previously associated with resistance, including *gacS*, *sigX* and *crfX* and two genes with no known function, were also mutated in some isolates with reduced antibiotic susceptibility. Our results show that exposure to sub-inhibitory amounts of antibiotics can select for mutations that reduce the susceptibility of *P. aeruginosa* to antibiotics and that the profile of mutations is different from that arising during selection with inhibitory antibiotic concentrations. It is likely that exposure to sub-inhibitory amounts of antibiotics during infection contributes to *P. aeruginosa* becoming antibiotic-resistant.

## Introduction

*Pseudomonas aeruginosa* is a widespread environmental bacterium that commonly infects the lungs of individuals with cystic fibrosis (*CF*) and other forms of lung disease (Garcia-Clemente et al., 2020). By adulthood, the majority of individuals with *CF* will have chronic *P. aeruginosa* respiratory infections (Smith et al., 2017; Malhotra et al., 2019). Acquisition of *P. aeruginosa* is associated with increased occurrence of sudden worsening of pulmonary symptoms (exacerbations), accelerated rate of lung function decline and decreased quality of life and reduced life expectancy (Elborn, 2016; Malhotra et al., 2019; Garcia-Clemente et al., 2020). Ongoing treatment with a range of antibiotics is used to improve lung function and limit the number of pulmonary exacerbations related to *P. aeruginosa* infections (Doring et al., 2012; Langan et al., 2015; Smith et al., 2018). The most commonly used antibiotics include aminoglycosides, such as tobramycin that inhibit protein synthesis, fluoroquinolones, such as ciprofloxacin that inhibit DNA synthesis, and β-lactams, such as ceftazidime and meropenem that inhibit cell wall synthesis (Smith et al., 2017). Although antibiotics are administered in amounts sufficient to inhibit bacterial growth *in vitro*, antibiotic effectiveness can be reduced within the infected airways due to a range of pharmacokinetic and pharmacodynamic factors including lung damage, blockage of airways and consequent poor oxygenation, co-morbidities and additional treatments that alter absorption and bioavailability of antibiotics (Akkerman-Nijland et al., 2021). These factors, combined with the tendency of *P. aeruginosa* to form biofilms in which the bacterial cells, are encased in a polysaccharide matrix, reduce antibiotic access to the target bacteria and potentially result in exposure of infecting *P. aeruginosa* to sub-lethal doses of antibiotics (Rogers et al., 2011; Maurice et al., 2018; Malhotra et al., 2019; Akkerman-Nijland et al., 2021). Exposure of bacteria to sub-lethal amounts of antibiotic has potential to result in the emergence of antibiotic resistance (Wistrand-Yuen et al., 2018).

Once long-term infections are established, antibiotic treatment is rarely sufficient to eradicate *P. aeruginosa* from the airways of individuals with *CF.* During long-term infections, *P. aeruginosa* becomes increasingly antibiotic-resistant, enhancing its survival (Sherrard et al., 2014; Smith et al., 2017). To better understand the genetic changes that lead to resistance, several research groups have exposed antibiotic-sensitive reference strains of *P. aeruginosa* to lethal concentrations of antibiotics and selected resistant mutants. Whole-genome sequencing (WGS) of the mutants identified the underlying resistance-causing mutations [Feng et al., 2016, Barbosa et al., 2017; Jorth et al., 2017; Melnyk et al., 2017; Yen and Papin, 2017; Sanz-Garcia et al., 2018a,b; Wardell et al., 2019; reviewed in (Lopez-Causape et al., 2018a)]. Mutations typically arose in genes that had previously been associated with resistance in clinical settings, such as *oprD* (carbapenem resistance) and *gyrAB* (fluoroquinolone resistance), and also in previously unrecognised resistance-associated genes, such as *ftsI* (carbapenem resistance) and *fusA1* (aminoglycoside resistance).

However, much less is known about the effects of low-level sub-inhibitory amounts of antibiotics on the genome of *P. aeruginosa* even though the bacteria likely encounter this situation during chronic infection. There is evidence that sub-lethal doses of antibiotics can increase the antimicrobial resistance of *P. aeruginosa* (Jorgensen et al., 2013; Wright et al., 2013; Ahmed et al., 2018, 2020; Moore et al., 2021). However, these studies have largely been limited to use of a single antibiotic (ciprofloxacin or in one case tobramycin) in conjunction with reference strains of *P. aeruginosa* rather than isolates from chronically infected patients. There has also been only limited investigation of the mutations arising following exposure of bacteria to sub-inhibitory amounts of antibiotics.

Fully understanding the effects of sub-lethal amounts of antibiotics on *P. aeruginosa* is of high clinical importance as incorrect treatment may facilitate emergence of antibiotic-resistant *P. aeruginosa* within the airways. Consequently, the aims of this research were to determine how often exposure to antibiotics at sub-lethal concentrations increases the amount of antibiotic that can be tolerated by these bacteria and to identify genetic changes associated with any changes in resistance phenotype.

## Materials and Methods

### Isolates of *P. aeruginosa*

Two well-characterised reference strains (PAO1 from our laboratory collection and PA14, kindly provided by Dr. Andrea Battistoni [University of Rome Tor Vergata, Rome, Italy]; Stover et al., 2000; Lee et al., 2006) and isolates of *P. aeruginosa* from sputum samples of six adults with *CF* (Table 1), collected under the approval of the New Zealand Health and Disability Ethics Committees (NTY/10/12/106), have been described previously (Martin et al., 2018; Freschi et al., 2019; Wardell et al., 2021). The selected isolates were sensitive to all of the antibiotics used in this study.

**Table 1:** Demographic details of *Pseudomonas aeruginosa* isolates obtained from people with cystic fibrosis.

						MIC (μg/mL)^*^			
Participant code	Gender	Age at time of isolate collection	Date of collection	Isolate MLST	Genome accession^†^	Ceftazidime	Ciprofloxacin	Meropenem	Tobramycin
S2239_16	M	26	Dec 91	244	SAMN0742408	0.75	0.84	0.38	0.27
DUN-003B	F	21	14 Aug 12	(no match)	SAMN07424128	0.50	0.16	0.063	0.30
DUN-009B	M	26	17 Oct 12	395	SAMN07424133	0.75	0.05	0.032	0.75
DUN-012-2	F	25	27 Feb 13	155	JAIPUJ000000000	1.0	0.05	0.094	0.50
DUN-015A	M	28	12 Dec 12	499	SAMN07424137	1.5	0.75	1.0	4.0
DUN-036-1	F	31	03 Mar 16	1,094	SAMN20982200	0.13	0.75	0.03	1.0
Reference strains									
PAO1				549	SAMN11606715	0.90	0.09	0.45	0.38
PA14				253	SAMN20982208	0.88	0.09	0.32	0.33

* *Determined by Etest. Values are averages of between 1 and 5 biological replicates (Supplementary Table S2)*.

† *NCBI Biosample number (https://www.ncbi.nlm.nih.gov/biosample/)*.

### Determination of Sub Inhibitory Concentration

Ciprofloxacin (Ciplox), ceftazidime and tobramycin (Mylan New Zealand Ltd) and meropenem (Penembact, Venus Remedies Limited) were used. Overnight cultures grown in Luria Bertani broth (LB broth; Miller, 1972) were adjusted to OD_600nm_ 0.01 and spot inoculated onto Mueller-Hinton (MH) agar plates (Difco) supplemented with doubling concentrations of antibiotic. The *SIC* was defined as being ¼ of the lowest antibiotic concentration that inhibited visible growth after overnight incubation at 37°C (Supplementary Table S1).

### Antibiotic Sensitivity Testing

Antibiotic sensitivity testing was carried out using ETEST strips (bioMérieux, North Ryde, Australia) in accordance with the manufacturer’s instructions. Briefly, bacteria from an overnight culture grown in LB broth were diluted to OD_600nm_ 0.1 in 0.9% normal saline (NaCl) and inoculated onto an LB agar plate. ETEST strips were added and plates incubated at 37°C for 18 h under aerobic conditions. The minimum inhibitory concentration (MIC) was determined at the point where the inhibition ellipse intersected the side of the antibiotic strip. In some cases, replicate tests were undertaken to confirm reproducibility.

### Antibiotic Passaging

Bacteria underwent 10 passages in the presence of antibiotic at *SIC* concentrations. In a subset of experiments, half or twice the *SIC* concentration was used. Briefly, a single colony inoculum in antibiotic-free LB broth was cultured overnight and adjusted to OD_600nm_ 0.1. An MH plate was surface flooded with a 750 μl aliquot of the prepared inoculum and excess culture removed. Following aerobic incubation (37°C, overnight), bacteria were harvested from the plate and used to inoculate antibiotic-free LB broth which was incubated overnight. This process was repeated for 10 passages. Four strains were also passaged 10 times under antibiotic-free conditions to assess any effect of passaging itself on changes to MIC or occurrence of genetic mutations.

### WGS and Bioinformatic Analysis

Genome assemblies for isolates S2239, DUN003B, DUN009B, DUN012-2 and DUN015A (Freschi et al., 2015) were generously provided by Prof Roger Levesque and co-workers. WGS of DUN036-1, the reference strains and passaged bacteria and identification of mutations was carried out as described previously (Wardell et al., 2019). Briefly, extracted DNA (MoBio UltraClean^®^ Microbial DNA isolation kit) underwent sequencing on the Illumina MiSeq platform with a minimum of 40-fold coverage across each genome. Raw sequence reads were trimmed using Trimmomatic version 0.36 (Bolger et al., 2014). Draft genome assemblies were created using SPAdes version 3.12.0 (Prjibelski et al., 2020) with the careful flag enabled. Draft genome assemblies were ordered relative to *P. aeruginosa* PAO1 using mauve version 2.4.0 (Darling et al., 2010) and then annotated using Prokka version 1.13 (Seemann, 2014). The resulting annotated draft genomes were used as a reference for mapping the sequence reads of passaged bacteria using Breseq version 0.35.0 (Deatherage and Barrick, 2014), allowing for contig reference (−c flag) and polymorphism mode enabled (−p flag). MLST types were determined from the genome sequences using pubMLST.^1^ For strain PA14, the reference genome was derived from the refseq genome sequence (NC 008463) using GDtools version 0.30 (Deatherage and Barrick, 2014) to incorporate sequence differences in the isolate used in this study. The genome of the reference strain PAO1 used in this study was previously derived from the refseq genome sequence (NC 002516) in the same way (Wardell et al., 2019).

### Data Availability Statement

All raw sequencing reads from mutants generated in this study are available under SRA BioProject PRJNA757894.

## Results

### The Effects of Sub-inhibitory Antibiotics on MIC

Five biological replicates of the laboratory reference strain PAO1 were passaged in the presence of sub-inhibitory concentrations (*SIC*) of each of four antibiotics. There were no significant changes in the MIC following passaging with tobramycin, ceftazidime or meropenem, where a significant change was defined as being a difference of more than one doubling dilution (> 2-fold; Brennan-Krohn et al., 2017; Table 2). In contrast, the MIC increased 4-fold or greater for four of five replicates passaged in the presence of *SIC* of ciprofloxacin.

**Table 2:** Changes in MIC and genetic variants identified following exposure to antibiotics at *SIC*.

Strain	Antibiotic	Biological Replicate	Fold change of MIC^*^	Non-synonymous mutations identified following WGS^c^
PAO1	Ceftazidime	1	1.0	
		2	1.0	
		3^a^	0.67	
		4^a^	0.67	
		5^a^	0.50	
	Ciprofloxacin	1	1.5	
		2^#^	6.8	*mexZ* (Δ11 bp, bp 321–331)*; pilB* (+2 bp, bp 276)
		3	4.0	*mexZ* (C113Y (TGC → TAC))
		4^^^	4.6	*nalC* (+1 bp, bp 31)
		5^^^	4.0	*fha*1 (Δ6 bp, bp 789–794)*; mexR* (Δ12 bp, bp 368–379)
	Meropenem	1	0.67	
		2	1.3	
		3	1.0	
		4	0.7	
		5	0.5	
	Tobramycin	1	2.0	
		2	1.0	
		3	2.0	
		4	1.3	
		5	1.5	
PA14	Ceftazidime	1	1.0	
		2	1.0	
	Ciprofloxacin	1^^^	1.2	
		2^^^	10.6	*mexS* (PA_32420; +21 bp, bp 891)
	Meropenem	1	0.7	
		2	1.0	
	Tobramycin	1	2.0	
		2^^^	2.0	
S2239_16	Ceftazidime	1	1.0	*mucA* (Δ42 bp, bp 329–371); Δ245581kb (PA2228 - *pvdL*)
		2	1.0	*mucA* (Δ42 bp, bp 329–371); no other detectable mutations
	Ciprofloxacin	1^†^	1.1	
		2^#^	0.9	
	Meropenem	1	2.0	
		2	2.0	
	Tobramycin	1^^^	2.8	*algU* (R174L (CGG → CTG)); *fusA1* (A603V (GCG → GTG))
		2^^^	2.4	*mucA* (Δ42 bp, bp 329–371); *ptsP* (Δ15bp, bp 1,436–1,452); *fusA1* (M461V (ATG → GTG))
DUN-003B	Ceftazidime	1	4.0	*sigX* (Q35H (CAG → CAT))
		2	1.5	
	Ciprofloxacin	1^#^,^b^	3.5	
		2^^^,^b^	2.0	*mucD* (Δ 21-bp, bp 296–318)
	Meropenem	1^^^	1.5	No detectable mutations
		2	2.0	*mucD* (Δ 21-bp, bp 296–318)
	Tobramycin	1^#^,^a^	2.5	*gacS* (L303H (CTC → CAC))
		2^^^,^a^	1.8	*glnE* (Q897* (CAA → TAA)); *argA* (G146C (GGC → TGC))
DUN-009B	Ceftazidime	1	1.0	
	Ciprofloxacin	1^^^	16.0	no detectable mutations
	Meropenem	1	0.7	
	Tobramycin	1	0.7	
DUN-012-2	Ceftazidime	1	0.5	
	Ciprofloxacin	1^^^	1.0	PA3921(F289S (TTC → TCC))
	Meropenem	1	0.5	
	Tobramycin	1	0.5	
DUN-015A	Ceftazidime	1	2.7	*crfX* (I59N (ATC → AAC))
	Ciprofloxacin	1	1.3	
	Meropenem	1	0.5	
	Tobramycin	1	1.0	
DUN-036-1	Ceftazidime	1	1.5	
	Ciprofloxacin	1	1.0	
	Meropenem	1^^^	6.3	PA2441 (P233H (CCC → CAC); PA4772 (+1 bp, bp 6)
	Tobramycin	1	2.0	

* *Fold change of MIC over time; Average value of up to three Etest replicates.*

^ Etest performed in replicate.

# Etest performed in triplicate.

† Etest performed in quadruple.

a Antibiotic concentration was half of *SIC*.

b Antibiotic concentration was double *SIC*.

c For substitution mutations, the amino acid change in the protein and the codon change in the gene is shown. For deletions (Δ) and insertions (+), the number of base pairs and the location within the gene is shown.

For comparison, we passaged biological duplicates of reference strain PA14 in the presence of *SIC* antibiotics. As with strain PAO1, there was no significant change in the MIC for ceftazidime, meropenem or tobramycin. However, there was an increase of 10.6-fold in the MIC in one of the duplicates passaged with ciprofloxacin.

To determine whether these findings were applicable to clinical isolates of *P. aeruginosa*, six susceptible isolates from respiratory infections of people with *CF* were passaged under *SIC* antibiotic conditions. The initial isolates studied, S2339_16 and DUN-003B, were tested in duplicate. In contrast to the reference strains, neither S2239_16 replicate showed a significant increase in MIC when passaged in the presence of ciprofloxacin, whereas there was a 3.5-fold increase for one of the DUN-003B replicates. In addition, there were slight increases in the MIC for tobramycin for each S2239_16 replicate (2.4- and 2.8-fold) and one replicate of strain DUN-003B (2.5-fold). One DUN-003B replicate had a 3.5-fold increase in the MIC following passaging in the presence of ceftazidime.

Passaging of an additional four isolates (DUN-009B, DUN-012-2, DUN-015A and DUN-036-1) was undertaken to further assess the impact of antibiotics at *SIC* levels on clinical isolates. Similar to the results for the initial two clinical isolates, exposure to antibiotics at *SIC* generally had no significant impact on the MIC. As exceptions, exposure to ceftazidime, ciprofloxacin and meropenem resulted in a 2.7–16- and 6.3-fold increase in the MIC for one replicate of DUN-015A, DUN-009B and DUN-036-1, respectively.

In total, 13 of the 60 passaged cultures had a significant (>2-fold) increase in MIC. Most of these were for ciprofloxacin (*n* = 7), followed by tobramycin (*n* = 3), ceftazidime (*n* = 2) and meropenem (*n* = 1). None of the increases in MIC were sufficiently high to cause the bacteria to be classified as clinically resistant (CLSI, 2018).

Passaging under antibiotic-free conditions was performed on a subset of strains and isolates (PAO1, PA14, DUN-003B and S2239_16), to determine whether passaging alone resulted in a change to MIC. None of the passaged bacteria had a significant change in MIC (Supplementary Table S3).

In a small number of experiments, passaging was carried out with half or double the *SIC*. In one of these experiments, half of the *SIC* resulted in an increased MIC for tobramycin in isolate DUN-003B. The MIC did not change in other experiments where passaging was carried out with different amounts of antibiotic (Table 2).

### The Effects of *SIC* on the Genome of *P. aeruginosa*

Bacteria that had a > 2-fold increase in MIC following passaging, and a selection of bacteria that did not, underwent WGS to identify any changes to the genome (Table 2, Supplementary Table S2).

All five of the reference strain replicates that had an increased MIC for ciprofloxacin had mutations in genes that control efflux pump activity (*mexZ*, *mexR, mexS* and *nalC* [Table 2]), consistent with the known role of efflux pumps in contributing to ciprofloxacin resistance (Rehman et al., 2019). No changes were identified in the only clinical isolate (DUN-009B) with an increased ciprofloxacin MIC after passaging. Failure to detect a mutation may have been because the passaged bacteria comprised a mixture of strain DUN-009B and a mutant with increased MIC. In a mixture, the mutant able to tolerate a higher concentration of antibiotic would be detected in MIC testing but a genetic mutation may go undetected, due to the presence of a high proportion of wild-type allele in WGS.

Passaged bacteria with an increased MIC for ceftazidime had mutations in the *sigX* gene or the *crfX* gene. So far as we are aware, these genes have not previously been associated with antibiotic resistance. A mutation in the *cmpX* gene, that is operonic with *crfX* and likely to have a related function, reduces expression of *sigX* (Bhagirath et al., 2018). Conversely, SigX upregulates the operon containing *cmpX* and *crfX* (Bouffartigues et al., 2020). These observations suggest a common pathway for increased ceftazidime MIC in the *crfX* and *sigX* mutants. SigX affects expression of genes encoding a variety of cellular functions including expression of the *oprF* gene that encodes an outer membrane porin (Bouffartigues et al., 2012) and genes involved in carbon metabolism, membrane fluidity, stress response and c-di-GMP signalling (Gicquel et al., 2013; Blanka et al., 2014; Flechard et al., 2018). How changes in SigX/CrfX alter susceptibility to ceftazidime is not yet clear.

Increased MIC following tobramycin exposure resulted in a range of mutations. Two of the three mutants with an increased MIC for tobramycin had mutations in *fusA1*, a known contributor to tobramycin resistance (Bolard et al., 2018; Lopez-Causape et al., 2018b; Scribner et al., 2020). One of these mutants also had a mutation in *ptsP* that has also been associated with tobramycin resistance (Feng et al., 2016; Sanz-Garcia et al., 2018b; Scribner et al., 2020). The third mutant had a mutation in the *gacS* global regulator gene, that controls a wide variety of cellular functions and intriguingly is influenced by the *cmpX* gene (Bhagirath et al., 2018) although its relationship with tobramycin resistance is not clear.

Only one mutant with an increased MIC was obtained following passaging of a clinical isolate in the presence of meropenem. This mutant had mutations in two genes, both of unknown function and neither of which have previously been associated with meropenem resistance.

Derivatives of clinical isolates S2239_16 and DUN-003B had mutations in genes (*mucA*, *mucD* and *algU*) associated with alginate biosynthesis following passaging in the presence of antibiotics. Clinical isolates frequently undergo changes in mucoidy that are associated with altered alginate production during subculture (Govan and Deretic, 1996). Indeed, two replicates of isolate S2239_16 passaged in the presence of ceftazidime had mutations in *mucA*, with no change in MIC (Table 2) and two replicates of the same isolate passaged in the absence of antibiotic also had mutations in *mucA* (data not shown). It is therefore likely that the *mucA* mutations are associated with adaptation of the bacteria to growth under laboratory conditions.

## Discussion

In chronic lung infection, the physiology of the airways, changes resulting from disease progression, and the biofilm growth of *P. aeruginosa* all inhibit delivery of antibiotics to the bacterial cells (Maurice et al., 2018; Akkerman-Nijland et al., 2021). It is therefore difficult to determine the dosage of antibiotics experienced by *P. aeruginosa* during infection in the airways of people with lung disease, but as antibiotics usually fail to clear established chronic infection, it is likely that the bacteria are exposed to *SIC* amounts. Furthermore, exposure to *SIC* amounts of antibiotic can increase bacterial ability to resist antibiotics (Wistrand-Yuen et al., 2018) and this likely causes the increased antibiotic resistance associated with prolonged infection (Sherrard et al., 2014; Smith et al., 2017). We therefore investigated the effects of prolonged antibiotic exposure at *SIC*. A total of 60 passaging experiments were carried out across four antibiotics and eight isolates of *P. aeruginosa*, and there was a significant increase in MIC in 13 of the passaged lines, showing that *SIC* exposure can increase ability of the bacteria to tolerate antibiotics. This finding is consistent with previous studies that indicated that exposure to antibiotics at *SIC* can impact the amounts of antibiotic that can be tolerated by *P. aeruginosa* (Jorgensen et al., 2013; Wright et al., 2013; Ahmed et al., 2018, 2020; Moore et al., 2021).

The effects of antibiotic exposure varied between isolates and between antibiotics. With the reference strains PAO1 and PA14, only ciprofloxacin exposure resulted in higher MIC, whereas across the 6 clinical isolates, all four antibiotics resulted in at least one derivative with a higher MIC. This difference may be the result of genetic or biological differences between isolates from people with *CF* and the reference strains PAO1 and PA14 that were initially derived from acute infections. Sub-MIC antibiotics can induce stress responses that increase the frequency of mutations (Blazquez et al., 2018), and stress responses may vary between isolates, and with different antibiotics, influencing the frequency of mutations. MIC has been reported to increase following exposure of strain PAO1 to *SIC* ciprofloxacin (Ahmed et al., 2018, 2020), of isolates from *CF* to *SIC* tobramycin (Moore et al., 2021), and of epidemic strain LESB58 to a range of antibiotics (Wright et al., 2013). More extensive analysis is required to determine whether there is a genuine difference between the clinical isolates and the PAO1 and PA14 reference strains. The strains and isolates used in this study were sensitive to all of the antibiotics, and none of the mutants that arose during passaging had MICs that would be classified as being resistant (CLSI, 2018). More extensive passaging, including passaging of the mutant bacteria generated here, may well lead to clinical resistance (Jorgensen et al., 2013; Moore et al., 2021).

Whole-genome sequencing identified genetic changes underlying increases in MIC. Mutations had occurred in a number of genes associated with antibiotic resistance. For example, mutations in the *fusA1* gene are frequently associated with tobramycin resistance (Bolard et al., 2018; Wardell et al., 2019). Mutations also occurred in genes *mexR* and *nalC* that encode direct and indirect regulators of the MexAB-OprM efflux pump (Poole et al., 1996; Cao et al., 2004), *mexZ* that encodes a repressor of the MexXY-OprM efflux pump (Morita et al., 2012) and *mexS* that encodes a putative oxidoreductase that influences production of the MexEF-OprN efflux pump (Morita et al., 2015). Overexpression of MexAB-OprM reduces susceptibility to ciprofloxacin and β-lactam antibiotics, overexpression of MexXY-OprM to aminoglycosides and fluoroquinolones and overexpression of MexEF-OprN to fluoroquinolones (Lopez-Causape et al., 2018b).

A number of passaged bacteria had mutations in *mucA*, *mucD* or *algU*, genes that are associated with the production of alginate and the development of mucoidy, a hallmark of chronic infections (Govan and Deretic, 1996). Others have also reported that mutations in *algU* are associated with altered alginate production and biofilm architecture and a decrease in susceptibility of *P. aeruginosa* to aminoglycosides (Hentzer et al., 2001). However, it should be noted that mutations arose in *mucA* in isolate S2239_16 that did not result in an increased MIC and were likely selected as part of the passaging process, independent of the presence of antibiotic.

A number of mutants with increased MICs had mutations in genes not so clearly associated with resistance. One mutant with reduced susceptibility to ciprofloxacin had a *pilB* gene mutation. Ciprofloxacin-resistant *P. aeruginosa* can have mutations in pilin-encoding genes (Wardell et al., 2019; Ahmed et al., 2020), although how pilin gene mutations reduce susceptibility to ciprofloxacin is not known. Mutations in the *sigX* gene and in the *cfrX* gene, a potential regulator of *sigX* expression, were present in mutants arising from ceftazidime exposure. SigX contributes to a wide range of cellular properties (Bouffartigues et al., 2012; Gicquel et al., 2013; Blanka et al., 2014; Flechard et al., 2018) and how it might influence ceftazidime susceptibility is not known. Intriguingly, the SigX and AlgU sigma factors are both involved in expression of the operon containing the *crfX* gene (Bouffartigues et al., 2020) but it remains to be determined whether this connection extends to mechanisms of antibiotic susceptibility. Lastly, a mutant with increased MIC for tobramycin had a mutation in the *gacS* gene. This gene is associated with regulation of biofilm formation and control of virulence factor production (Valentini et al., 2018) but has not previously been associated with antibiotic resistance. How these genes contribute to antibiotic susceptibility phenotype will require further investigation.

Interestingly, several genes that are frequently mutated during experimental evolution in the presence of inhibitory amounts of antibiotics were not mutated following the *SIC* selection procedure. These include mutations associated with ciprofloxacin resistance, such as *gyr* and *par* gene mutations (Melnyk et al., 2017; Rehman et al., 2019). Similarly, resistance to meropenem and ceftazidime is often associated with mutations in the *oprD* and *dacB* genes, respectively (Lister et al., 2009; Chalhoub et al., 2016; Lopez-Causape et al., 2018b; Sanz-Garcia et al., 2018a; Wardell et al., 2019), mutations that did not arise in this study. Although relatively small numbers of mutants with increased MIC were analysed, these findings suggest that the selection that occurs during passaging with *SIC* amounts of antibiotic is different from that which occurs during passaging in lethal amounts of antibiotic. They also demonstrate that development of antibiotic resistance is complex and multifactorial.

While this study furthers the understanding of the antibiotic resistance of *P. aeruginosa*, there are some limitations. Firstly, inclusion of a larger panel of isolates would more strongly test the effects of *SIC* amounts of antibiotics. Secondly, isolates were passaged 10 times in the presence of *SIC* amounts of antibiotics and increasing the number of passages would likely have increased the proportion of bacteria with increased MIC. Lastly, sequencing of populations of passaged bacteria may have masked mutations that were present in a subset within a population. Growth of bacterial populations in the absence of antibiotic, during the passaging process, may also have reduced the proportion of bacteria containing any mutations that negatively affect fitness. Greater sequencing depth, or characterisation of multiple individual clones from each passaged population, may have identified more genetic changes.

In conclusion, our findings demonstrate that exposure to *SIC* levels of antibiotics can result in increased MIC of a range of *P. aeruginosa* strains and also identifies the underlying mutations. Many of the mutated genes have known roles in antibiotic resistance, although some of those in clinical isolates have not been previously associated with resistance. Prolonged exposure to *SIC* amounts of antibiotic during infection are likely to result in the emergence of antibiotic-resistant *P. aeruginosa*. Our findings emphasise the importance of achieving high antibiotic concentrations in the lungs of patients.

## Data Availability Statement

The datasets presented in this study can be found in online repositories (see Table 1). The names of the repository/repositories and accession number(s) can be found at: https://www.ncbi.nlm.nih.gov/, JAIPUJ000000000 https://www.ncbi.nlm.nih.gov/, PRJNA757894.

## Author Contributions

KR: data curation, formal analysis, funding acquisition, investigation, supervision, validation, writing – original draft, and writing – review and editing. SM: data curation, investigation, and writing – review and editing. SW: formal analysis and writing – review and editing. IL: conceptualisation, data curation, formal analysis, project administration, supervision, writing – original draft, and writing – review and editing. All authors contributed to the article and approved the submitted version.

## Funding

This work was supported by the Otago Medical Research Foundation (AG 374) and the Health Research Council of New Zealand (17/372). SW was supported by a Postgraduate Scholarship from the University of Otago.

## Conflict of Interest

The authors declare that the research was conducted in the absence of any commercial or financial relationships that could be construed as a potential conflict of interest.

## Publisher’s Note

All claims expressed in this article are solely those of the authors and do not necessarily represent those of their affiliated organizations, or those of the publisher, the editors and the reviewers. Any product that may be evaluated in this article, or claim that may be made by its manufacturer, is not guaranteed or endorsed by the publisher.

## Footnotes

^1^ https://pubmlst.org
