# Supplementary material for: The Effects of Sub-inhibitory Antibiotic Concentrations on Pseudomonas aeruginosa: Reduced Susceptibility Due to Mutations
Source: Front Microbiol. 2021 Dec 20;12:789550. doi: 10.3389/fmicb.2021.789550 (PMC8721600; doi:10.3389/fmicb.2021.789550)
Supplement: Supplementary file 1 [file Table_1.pdf]

Supplementary Table S1. Sub-inhibitory antibiotic concentrations (SIC) used for antibiotic exposure experiments.

		Ceftazidime		Ciprofloxacin		Meropenem		Tobramycin	
Pseudomonas aeruginosa isolate	Biological Replicate	IC*	SIC	IC	SIC	IC	SIC	IC	SIC
PA01	1	1†	0.25	0.125	0.032	0.5	0.125	0.5	0.125
	2	1	0.25	0.125	0.032	0.5	0.125	0.5	0.125
	3	1	0.125	0.125	0.032	0.25	0.063	0.25	0.063
	4	1	0.125	0.125	0.032	0.25	0.063	0.25	0.063
	5	1	0.125	0.125	0.032	0.25	0.063	0.25	0.063
PA14	1	1	0.25	0.125	0.032	0.25	0.063	0.5	0.125
	2	1	0.25	0.125	0.032	0.25	0.063	0.5	0.125
S2239_16	1	0.5	0.125	1	0.25	0.125	0.032	0.25	0.063
	2	0.5	0.125	1	0.25	0.125	0.032	0.25	0.063
DUN-003B	1	0.5	0.125	0.25	0.125	0.125	0.032	0.5	0.063
	2	0.5	0.125	0.25	0.125	0.125	0.032	0.5	0.063
DUN-009B	1	0.5	0.125	0.063	0.016	0.063	0.016	0.25	0.063
DUN-012-2	1	0.25	0.063	0.063	0.016	0.032	0.008	0.032	0.008
DUN-015A	1	0.5	0.125	0.5	0.125	1	0.25	1	0.25
DUN-036-1	1	0.032	0.008	0.25	0.063	0.032	0.008	0.032	0.008

*IC, the lowest antibiotic concentration that inhibited visible growth after overnight incubation, as determined by the doubling dilution method

†Antibiotic concentrations are in µg/mL
