# Supplementary material for: The Effects of Sub-inhibitory Antibiotic Concentrations on Pseudomonas aeruginosa: Reduced Susceptibility Due to Mutations
Source: Front Microbiol. 2021 Dec 20;12:789550. doi: 10.3389/fmicb.2021.789550 (PMC8721600; doi:10.3389/fmicb.2021.789550)
Supplement: Supplementary file 3 [file Table_3.pdf]

Supplementary Table S3: Minimal inhibitory concentrations of antibiotics following passaging under antibiotic-free conditions.

		PAO1			PA14			S2239_16			DUN-003B		
	Biological Replicate	MIC P0	MIC P10	MIC fold change	MIC P0	MIC P10	MIC fold change	MIC P0	MIC P10	MIC fold change	MIC P0	MIC P10	MIC fold change
Ceftazidime	1	1.0	1.0	1.0	0.75	1.0	1.3	0.75	0.75	1.0	0.5	0.5	1.0
	2	1.0	1.0	1.0	1.0	1.0	1.0	0.75	0.75	1.0	0.5	0.75	1.5
Ciprofloxacin	1	0.064	0.064	1.0	0.064	0.064	1.0	0.5	0.75	1.5`	0.125	0.19	1.5
	2	0.064	0.094	1.5	0.064	0.094	1.5	0.5	1.0	2.0	0.125	0.094	0.75
Meropenem	1	0.38	0.38	1.0	0.38	0.19	0.5	0.38	0.38	1.0	0.032	0.047	1.5
	2	0.50	0.50	1.0	0.25	0.38	1.5	0.38	0.38	1.0	0.094	0.125	1.3
Tobramycin	1	0.38	0.50	1.3	0.38	0.38	1.0	0.19	0.19	1.0	0.25	0.25	1.0
	2	0.38	0.38	1.0	0.19	0.38	2.0	0.38	0.38	1.0	0.19	0.19	1.0

Abbreviations: MIC, minimum inhibitory concentration (μg/mL) determined using eTest; P0, MIC prior to passaging; P10AB-, MIC following ten passages under antibiotic-free conditions.
